# Improving surface and defect center chemistry of fluorescent nanodiamonds for imaging purposes—a review

**DOI:** 10.1007/s00216-015-8849-1

**Published:** 2015-07-29

**Authors:** Andreas Nagl, Simon Robert Hemelaar, Romana Schirhagl

**Affiliations:** University Medical Center Groningen, Groningen University, Antonius Deusinglaan 1, 9713 AW Groningen, The Netherlands

**Keywords:** Diamonds, Surface chemistry, Sensors, Magnetometry, Biolabels

## Abstract

Diamonds are widely used for jewelry owing to their superior optical properties accounting for their fascinating beauty. Beyond the sparkle, diamond is highly investigated in materials science for its remarkable properties. Recently, fluorescent defects in diamond, particularly the negatively charged nitrogen-vacancy (NV^-^) center, have gained much attention: The NV^-^ center emits stable, nonbleaching fluorescence, and thus could be utilized in biolabeling, as a light source, or as a Förster resonance energy transfer donor. Even more remarkable are its spin properties: with the fluorescence intensity of the NV^-^ center reacting to the presence of small magnetic fields, it can be utilized as a sensor for magnetic fields as small as the field of a single electron spin. However, a reproducible defect and surface and defect chemistry are crucial to all applications. In this article we review methods for using nanodiamonds for different imaging purposes. The article covers (1) dispersion of particles, (2) surface cleaning, (3) particle size selection and reduction, (4) defect properties, and (5) functionalization and attachment to nanostructures, e.g., scanning probe microscopy tips.

**Graphical Abstract:**
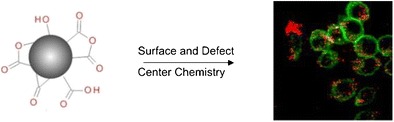
We review how diamond surface and defect chemistry can be optimized for different (bio) applications

We review how diamond surface and defect chemistry can be optimized for different (bio) applications

## Introduction

Owing to their outstanding properties diamonds have attracted the attention of scientists in many different fields. Besides their traditional use as jewels or abrasives, diamonds have many applications in modern scientific disciplines.

Owing to their inertness and excellent electrical properties (hydrogen-terminated diamond is surface conductive, boron-doped diamond is a semiconductor), they are widely used as transducers in chemical sensing [1–3]. To utilize diamond in imaging applications, fluorescent nanodiamonds are required. The fluorescence is caused by lattice defects in the diamond material. Among the around 700 different defects in diamond the negatively charged nitrogen-vacancy (NV^-^) center is by far the most prominent because of its outstanding properties; thus, this article focuses on this defect [4]. However, finding new defects and studying their properties is an actively researched area, and future studies might reveal other interesting defects. So far the most notable other defect is the silicon-vacancy (Si-V) center [5–7].

The NV^-^ center is a fairly stable single quantum emitter that can be controlled and read out optically This property, together with the excellent inertness of diamond, qualifies it as a promising candidate for quantum information and storage devices [8–10]. Nanodiamonds are interesting for several reasons. Owing to their high biocompatibility and inertness, they are being investigated as coating material for implanted medical devices [11–14]. They are used as an additive in sunscreens and other cosmetical products [15]. Another application is drug delivery. Owing to their small size and biocompatibility, nanodiamonds can be used as vehicles for drugs to enter cells [16–20]. Furthermore, the solubility of drugs in body fluid could be increased by their binding to nanodiamond [21]. In this review we focus on applications of fluorescent nanodiamonds in imaging. We discuss the demands on the diamond material and how the material can be improved.

Imaging applications involving diamond can be divided into two main types: applications based on fluorescence imaging only (see “Biolabels” and “Scanning FRET”) and methods based on the spin properties of the defect (see “Magnetometry applications”). Since the requirements for the surface chemistry differ between these two types, this distinction is also reflected in the structure of this article. We consider the requirements for the diamond material used as biolabels in “Demands on the diamond material.” Since nanodiamonds do not bleach, they would be an attractive replacement for organic dyes for such applications. Over other emitters such as quantum dots (which have similar properties) they have the striking advantage that toxicity is a much smaller issue (see also “Limitations for nanodiamonds in biolabeling”) [22]. Another application we discuss is scanning Förster resonance energy transfer (FRET) [23, 24] (see “Scanning FRET”). This technique is used to determine distances between a fluorescent donor and a quencher. Owing to their stable fluorescence defects, diamonds are a promising replacement for dye molecules that are regularly used for this purpose.

We dedicate “Surface chemistry” to one of the major limitations for using nanodiamonds for these applications: their varying surface chemistry (surface chemistry aspects specific to magnetometry applications are considered in “Surface chemistry”). We review recent advances and compare methods to alter surface chemistry as well as defect center chemistry of nanodiamond specific for these applications and give a practical guide to improve diamond quality. Although there are excellent reviews on the physical properties of diamond and special applications, there is no recent review specifically on the application-specific issues for surface chemistry.

In “Magnetometry applications,” we discuss diamond magnetometry as the most prominent method based on the spin properties of the defect. NV^-^-center-based magnetometers are currently among the most sensitive magnetic sensors available [25]. However, the sensitivities that were theoretically predicted to be the limit have not yet been achieved [26]. A related topic we will discuss is the use of nanodiamonds for sensing unpaired spins in the environment. Unlike other sensors that monitor changes in the environment, nanodiamonds can be very close or even inside a living cell.

Although our focus is on the use of nanoparticles, we will also mention a few methods that were investigated for bulk diamond since they are applicable to nanodiamonds as well.

## Diamond starting materials

There are several different sources of nanodiamonds [27]. The smallest members of the diamond family are known as diamondoids, and are typically extracted from crude oil [28]. Lower diamondoids such as adamantane are available via synthesis, whereas synthesis of higher diamondoids still poses problems [29]. Dahl et al. [30] have demonstrated the production of microcrystalline diamond in a chemical vapor deposition reactor using diamondoids as a precursor. They propose a chemical-vapor-deposition-type diamond growth mechanism in oil and gas fields. However, their diamondoids usually have a size of only around 1 nm. Although there were some attempts to incorporate defects into diamondoids, these are generally too small to allow there to be stable defects in sufficient quantities.

Detonation nanodiamonds are synthesized by the controlled detonation of TNT-like explosives [31]. They consist of a diamond core surrounded by a few layers of *sp*^2^ carbon and typically have a size of 4–5 nm. The stability and surface termination of these types of nanodiamonds are covered in the theoretical work of Raty and Galli [32] and Kaviani et al. [33], highlighting interesting aspects of the size distribution and the smallest possible sizes of nanodiamonds. However, their small size and relatively high amounts of impurities pose a problem for magnetometry or quantum science applications, and their low brightness is unfavorable for biolabeling applications. Thus, the above-mentioned diamond materials are not discussed further in this article.

A much purer alternative can be obtained by grinding large crystals to sub-100-nm-sized particles. The smallest commercially available nanodiamonds have median particle sizes as low as around 15 nm, and particles as small as around 5 nm in diameter have been produced by research initiatives [34, 35]. Unprecedented nanodiamond quality in terms of coherence times can be reached by nanofabrication of high-purity bulk diamonds [36]. Furthermore, not only the size but also the shape can be tailored to some extent. The drawback of this method is the relatively low amount of material obtained.

## Biolabels

**Fig. 1:**
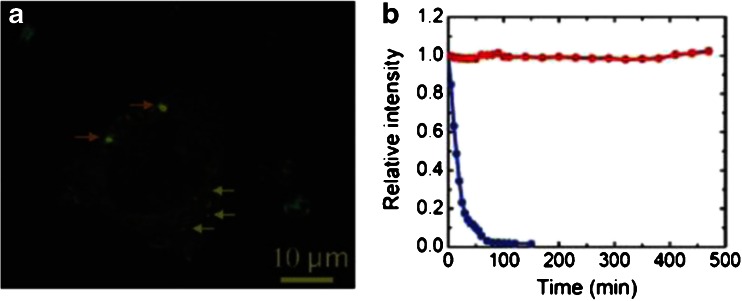
**a** A confocal microscopy image of a cell that has taken up fluorescing nanodiamonds. **b** The stable fluorescence of the negatively charge nitrogen-vacancy (NV^-^) center compared with the fluorescence of a commonly used fluorescent dye. (**a** Reprinted with permission from [56]; **b** reprinted with permission from [55])

Fluorescence labeling is one of the most important and most powerful methods in molecular biology [37]. The standard approach is to attach a fluorescent molecule to an antibody which selectively binds the target structure [38, 39]. A common alternative is to use the strong and selective binding between biotin and streptavidin [40]. While one of them is attached to a label, the other one is targeted. However, fluorescent labels have a major limitation: The fluorescence is bleached over time [41–43]. In contrast, defects in diamond stably fluoresce [44, 45] over very long times, which allows averaging and thus an increase in resolution [46]. Figure 1b shows a comparison between the stable fluorescence of an NV^-^ center and the bleached fluorescence of a commonly used fluorescent dye. This is a property that fluorescent diamonds have in common with quantum dots [47–49]. Although traditional semiconducting quantum dots are usually toxic at least to some extent [50–52], there are several studies showing low or no toxicity for diamond [53–55]. Thus, it is promising to use diamond particles as a substitute for fluorescent dyes in labeling. Figure 1a shows a confocal microscopy image where this was realized with fluorescent diamonds incorporated into a cell [56]. For recent reviews of special applications of diamond labeling, we refer the reader to [57] for diagnostics, [31, 58] for detonation nanodiamonds for biological applications, [59] for biomedical applications, [60] for biological applications in general, and [61] for applications in physics and biology.

### Demands on the diamond material

**Fig. 2:**
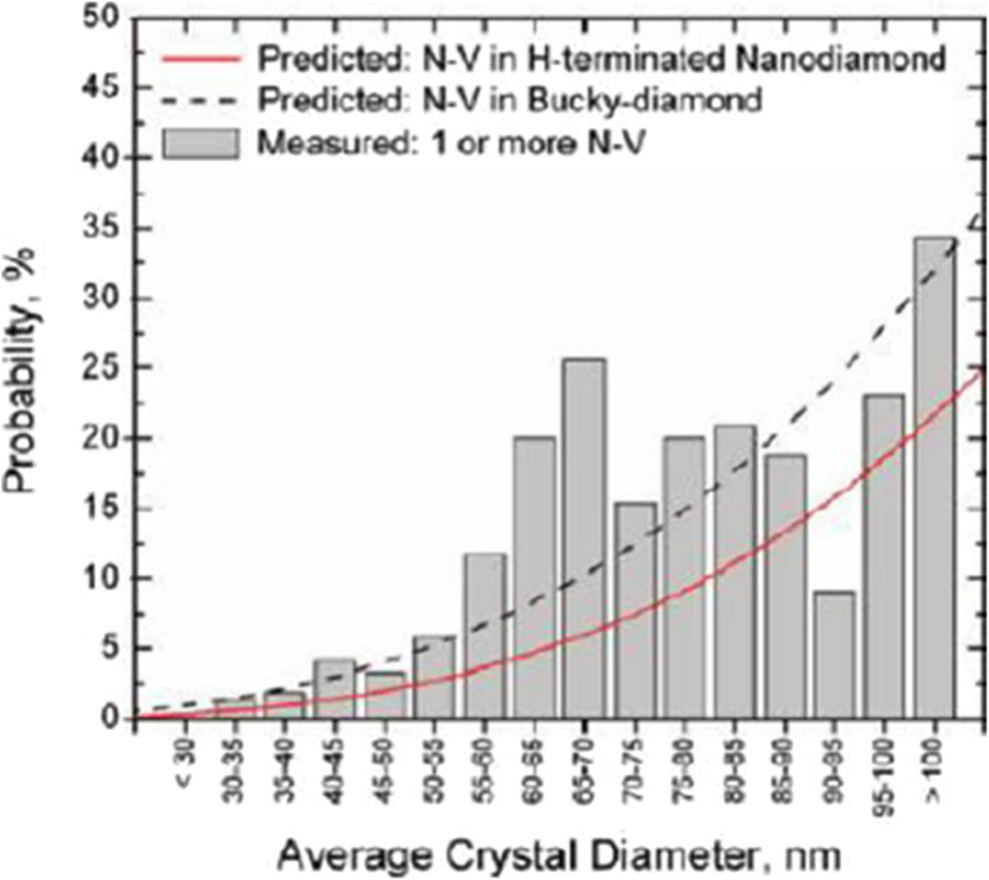
Correlation between diamond size and the number of NV^-^ centers in a nanodiamond. (Reprinted with permission from [65])

There are several requirements for diamond material that are specific to biolabeling applications. For in vivo labeling, diamond particles need to be smaller than around 200 nm in order to penetrate the cell membrane [62]. This size might be different depending on the cells, the particle, and the application. Macrophages, for example, are easier to target since they naturally phagocytose particles. A generally interesting method was described by Smith et al. [63]. They incorporated diamond into lipid micelles to ease cellular uptake. Thus, they were able to bring diamonds into mammalian epithelial cells. If the diamond particle should also penetrate the nucleus, it needs to be even smaller (30–40 nm) [64]. The probability of finding a stable defect (decreasing with the size of the diamond) confines the smallest sizes that are used for biolabeling [65]. Figure 2 shows the probability of finding a defect depending on the diamond particle size.

Thus, as high as possible brightness is preferred for biolabeling. Alternatively, utilization of the photoactive Si-V color center might give access to diamond nanoparticles smaller than 10 nm, thus possibly allowing better biological imaging [45]. Even particles as small as 1.6 nm are reported to be capable of hosting stable Si-V centers [66]. However, for measurements making use of the NV^-^ center, generally two strategies exist: increasing brightness by artificially enhancing the number of NV^-^ centers per diamond (discussed in “Engineering defects”) or differentiating the NV fluorescence from the background [67]. In addition to using filters (which also subtract the excitation wavelength), an elegant approach was used by Igarashi et al. [67]. They used microwave pulses to differentiate the NV fluorescence from the autofluorescence from biological samples (only signals that change when the samples were subjected to microwave pulses were assigned as an NV^-^ signal; the principle is explained in “Scanning FRET”).

### Surface chemistry for biolabels

To use nanodiamond as a biolabel, one has to attach a selective biomolecule. The easiest way to achieve this is by simple physisorption of biomolecules on the diamond. However, if a more controlled or more permanent attachment is desired, one can also covalently attach molecules. Although diamond is generally known to be very inert, a surprisingly high number of reactions have successfully been performed on diamond surfaces. Often, a three-step process is used: The first step is a relatively harsh treatment to introduce functional groups on the surface. This also increases homogeneity of the surface. (Generally, hydrogen-terminated diamond is more homogeneous than oxygen-terminated diamond and thus is often the intermediate of choice [68].) Once functional groups are in place, they can be used to bind different linker molecules to the surface in a second step. In a final step, all kinds of biomolecules can be attached to the linker. Importantly, this step can be performed under mild and biocompatible conditions (depending on the application, biomolecules can also be attached in fewer steps if, e.g., the molecule binds directly to the functional groups introduced in the first step [69]).

**Fig. 3:**
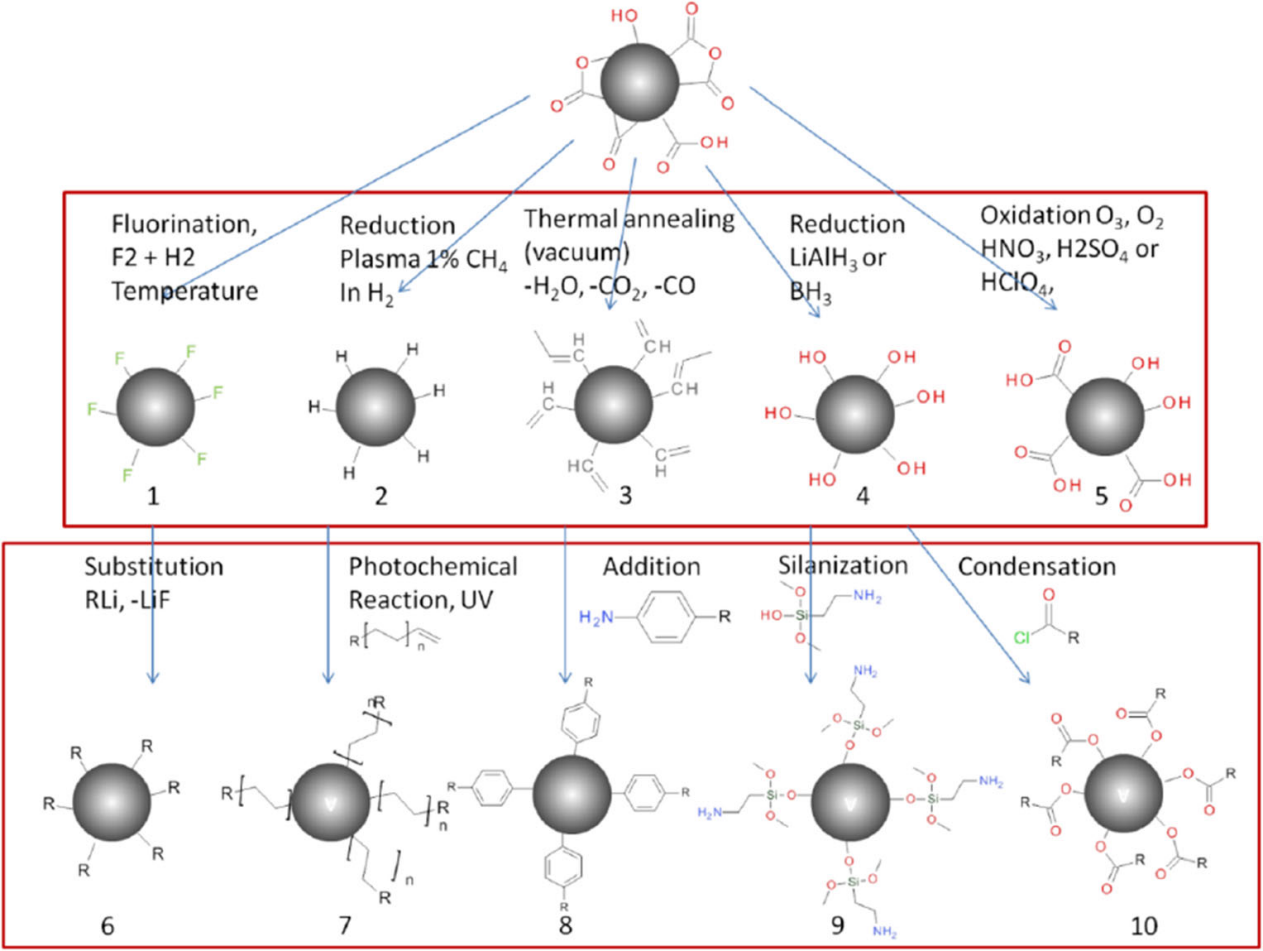
Overview of the most important surface modification methods for covalently attaching molecules to diamond: The *upper half* shows different first steps that are performed to provide a homogeneous surface. Details on the synthesis for the different surface terminations can be found in the respective references: *1* [71], *2* [174, 175], *3* [176, 177], *4* [71], *5* [178]. The *lower half* shows different ways of attaching a linker molecule (*R* stands for the desired functional groups, e.g., NH_2_): *6* [74], *7* [174, 175], *8* [179], *9* [71, 72], *10* [73]

An overview of the most important methods is given in Fig. 3. Different first steps that have been used include the following. Oxidative treatment of diamonds leads to the formation of carboxyl groups, which can then react with alcohol or amine derivatives [70]. Another approach, reported by Krueger et al. [71], is to reduce all oxygen-containing surface groups to OH functions with borane, then allowing the grafting of a variety of silanes [72] or long alkyl chains [73]. Halogenation has also been reported, such as thermal fluorination [74–76] and plasma fluorination [77]. Fluorinated diamonds can then react with nucleophilic (as lithium organic compounds) reagents in substitution reactions leading to amino or acid terminations.

Then a linker molecule is attached that has a functional group that can bind to different biomolecules. Attaching possible linkers for the respective starting materials is illustrated in the lower half of Fig. 3. Finally, a biomolecule, which provides the selectivity, is attached to the linker. Antibodies, biotin/streptavidin, aptamers, and DNA are possible candidates for targeting biomolecules. More details on attachment methods are given in the next sections.

#### Antibodies

**Fig. 4:**
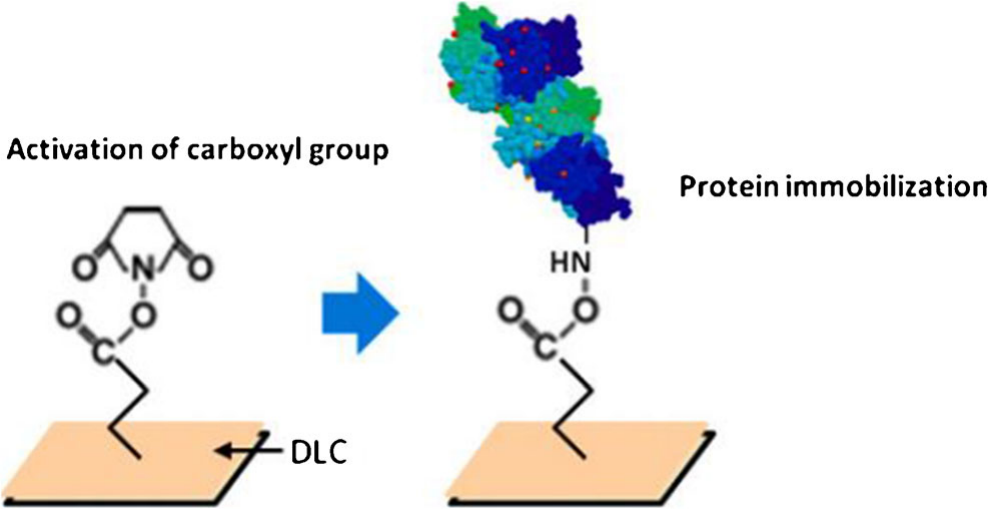
Attaching a protein, e.g., an antibody, to a diamond surface. *DLC* diamond like carbon. (Reprinted with permission from [82])

Glycoproteins that selectively bind a certain target are the most commonly used biomolecules in fluorescent labeling [78, 79]. The easiest way of attaching antibodies to diamond is by simple physisorption [80, 81]. The surface of hydrogen-terminated diamond is slightly positively charged and attracts antibodies that are, as most proteins, negatively charged. Suzuki et al. [82] used a method to covalently attach a protein to a diamond surface. They used approach 1 to 6 in Fig. 3 to attach *N*-hydroxysuccinamide (NHS; the molecule shown on the left in Fig. 4). This is a standard molecule for attaching proteins to a surface. It provides an excellent leaving group that can be substituted by amine groups of proteins.

#### Biotin/streptavidin

The interaction between streptavidin and biotin is one of the strongest noncovalent interactions in nature and is thus widely used for labeling [83–85]. Krueger et al. [72] discovered a method to covalently attach biotin to surface-functionalized aggregates of detonation nanodiamonds. To this end, they used pathway 4 to 9 in Fig. **3**. The biotin molecule was attached by an amide bond between the carboxylic acid group of biotin and the amine moiety provided by the silane linker. They performed binding tests for streptavidin after attachment, and found that biotin was still able to bind streptavidin. Marcon et al. [87] used a method (discovered by Boukherroub et al. [86]) to covalently attach streptavidin to a diamond surface. They used UV irradiation to provide surface 5 in Fig. **3**. Then they photochemically attached benzophenone moieties to generate carbonic acid groups on the surface. These groups were then used to covalently attach streptavidin.

#### Aptamers

Aptamers are small nucleic acid sequences that selectively bind to target molecules, and can be seen as a nucleic acid analogue of antibodies [88, 89]. Kuga et al. [90] investigated a method to attach aptamers to diamond. To generate a homogeneous surface (2 in Fig. **3**), they exposed the diamond to hydrogen plasma. Amination of hydrogen-terminated diamond was performed by irradiation with UV light at low pressure at the presence of ammonia gas. The aptamer was attached by covalent bonding to an NHS linker. They used their method for determining DNA mismatches. A similar approach was used for sensing platelet-derived growth factor by other authors [91–93]. Tran et al. [94] attached an aptamer to a diamond surface that recognizes immunoglobulin E. They used approach 3 (Fig. **3**) to provide a homogeneous surface and then photochemically attached unsaturated fatty acids. These have carboxyl groups that form amide bonds with immunoglobulin E.

#### Other molecules

If the target biospecies is, for instance, an antibody, the antigen can also be attached to diamond. Such an approach was used by Hartmann et al. [95], who immobilized saccharides on the surface of nanodiamonds. They used approach 3 to 8 in Fig. **3** to achieve this goal. These saccharides were recognized by specific receptors on the cell wall of bacteria. This way they selectively labeled different cell types. Krueger et al. [71] attached peptides to surface-modified detonation nanodiamonds. They used a reduced (OH groups) diamond surface and grafted silanes, which were used to attach peptides (pathway 4 to 9 in Fig. **3**). They observed some aggregation of diamonds during silanization; however, during later steps, the aggregate size was fairly stable and did not increase any further. Yang et al. [96] attached DNA to a diamond surface to target complementary DNA strands. They used method 2 to 7 in Fig. **3** to insert an NHS leaving group as shown in Fig. **4**. Instead of the protein that Suzuki et al. [82] used, they attached DNA by the same mechanism. Ushizawa et al. [70] also attached DNA to diamond particles. They used approach 4 to 10 in Fig. **3** to provide first a homogeneous surface chemistry and then an acid chloride moiety. The acid chloride group then formed an ester with an OH group of desoxyribose sugar (part of the DNA backbone). Fu et al. [97] attached polylysine via an NHS linker (analogue to Fig. **4**) to a diamond surface (carbonic acid terminated). Polylysine is a positively charged peptide that interacts nonspecifically with proteins and DNA. The system was used to investigate the interaction with HeLa cells (an immortal human cancer cell line). The same surface modification method was used by Cheng et al. [98], who performed 3D tracking of nanodiamonds inside HeLa cells. To increase the amount of NV^-^ centers, they used helium ion irradiation.

Fang et al. [99] reported an interesting labeling strategy that did not even require the attachment of molecules to the diamond surface that involved simply incubating a culture of HeLa cells with nanodiamonds. After the cells had phagocytosed the diamonds, the diamonds could be used for long-term fluorescence tracking of the cells. The diamonds were also found in daughter cells after cell proliferation.

#### Limitations for nanodiamonds in biolabeling

To date the most limiting factor for the use of fluorescent nanodiamonds in biology is the low brightness of the NV^-^ center. Compared with standard organic dyes, the emission of an NV^-^ center is low. If the diamond particle hosts only one or very few defects, very sensitive detection is necessary to see them. Furthermore, it is problematic to differentiate the particle from the background fluorescence. To circumvent this problem, diamonds containing many NV^-^ centers are used. However, this requires larger diamonds (tens of nanometers). This larger size also poses a problem for some bioapplications. Lastly, the toxicity of nanodiamonds is mostly unexplored (especially in vivo). Some (often very low) toxic effects have been found for detonation nanodiamonds with different terminations (owing to their small size their properties are largely determined by the surface) [100–104], whereas no toxicity has been reported by some authors[105]. Even some toxic effects of oxygen-terminated detonation nanodiamond on bacteria have been reported [106]. Generally, it has been shown that smaller (5-nm) carboxylated diamonds are slightly less biocompatible than larger diamonds (more than 100 nm) [107, 108]. This is most likely due to less inert surface material, and has been found to depend on the termination (differences in cell behavior on bulk diamond with different termination has also been demonstrated) [109]. For larger nanodiamonds, which are the topic of this review, no toxic effects have been identified so far [22, 53–55, 108]. However, data on this issue, which might be very different for various cell types or organisms, are sparse. Especially data on the long-term fate of nanodiamonds and if they are cleared from the body in organisms are lacking.

Additionally (as with every other label), care has to be taken to avoid structural changes of attached biomolecules. Perevedentseva et al. [110] have provided some evidence that bound nanodiamonds might alter the structure and function of attached proteins. They reported that this effect was more pronounced for detonation nanodiamonds than for bigger microcrystalline diamond. This could be caused by the rich surface chemistry of detonation diamonds rather than the diamond material itself.

## Scanning FRET

Another imaging method where diamond has a high potential is scanning FRET. FRET is a powerful technique for the study of intermolecular interactions. It is based on dipole–dipole coupling, through which an electronically excited donor fluorophore transfers its excitation energy to a nearby acceptor fluorophore, resulting in changes in both the fluorescence intensity and the lifetime of the donor. This method is very useful for determining distances of small molecules [111] or for monitoring interactions [112]. The NV^-^ center is a promising candidate for a donor owing to its photostability and relatively long lifetime. Boersch et al. [113, 114] proposed the use of fluorescent nanodiamonds as donors for FRET analysis of the rotation of molecular motors. They were particularly interested in the ATP synthase motor, which is embedded in the cell membrane. The diamond is located on the static parts of the motor, and Boersch et al. suggest an acceptor is located on the rotor. Possible partners are organic-dye-based quenchers in the near-infrared region. Depending on the orientation of the rotor, the distance between the donor and the acceptor will vary, which leads to changes in fluorescence. A first proof-of-principle experiment was done by Tisler et al. [115]. They monitored the FRET signal of nanodiamonds with covalently attached acceptor molecules. When acceptor molecules were bleached, less FRET interaction was observed, resulting in an increase in fluorescence. Chen et al. [116]. incorporated both diamond and an acceptor into a polymer matrix, and they also bleached some of the dye molecules and monitored the increase in fluorescence.

The major drawback of using diamonds for FRET analysis is their relatively large size. Care has to be taken that the diamond does not influence the natural behavior of the system too much. Additionally, the smallest distances that can be measured are also limited by the size of the diamond. Since the defect typically is a few nanometers from the surface, this defines the minimum distance to its FRET partner. Additionally, the low brightness might pose a problem here as well.

## Magnetometry applications

**Fig. 5:**
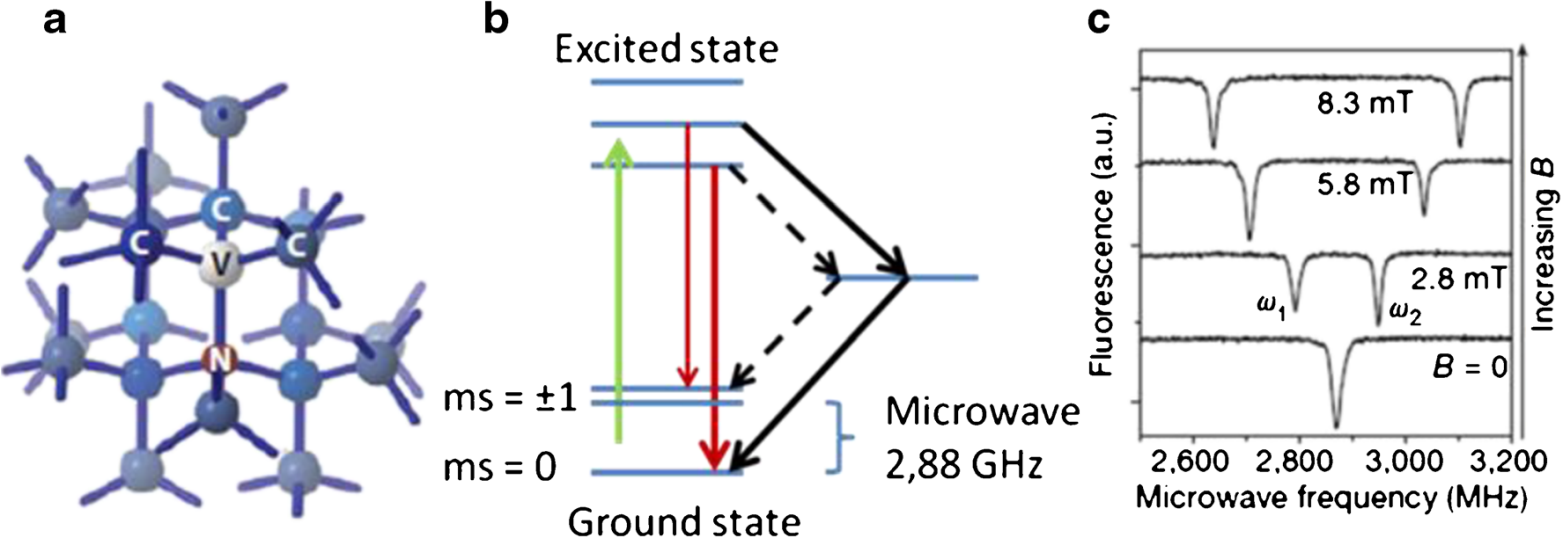
Optomagnetic properties of the NV^-^ center. **a** One carbon atom is replaced by a nitrogen next to a vacancy in the diamond lattice. **b** Simplified energy diagram of an NV^-^ center. After excitation with a green laser, the NV^-^ center emits red photons. If the electron is in the *m*_*s*_ = ±1 state, there is also an alternative way to the ground state over a dark state. As a result, fewer red photons are emitted and decreased fluorescence is observed. If a microwave is applied whose energy equals the difference between the energies of the two states (2.88 GHz at zero field), the spins flip into the *m*_*s*_ = ±1 state. This effect can be observed as a drop in fluorescence (*bottom curve* in **c**). In presence of an external magnetic field the *m*_*s*_ = ±1 states are no longer equal in energy and thus split into two lines (*top three curves* in **c**). The difference is proportional to the field (Zeemann splitting), and the magnetic field can be determined. (**a** Reprinted with permission from [27]; **c** reprinted with permission from [164])

Detection of weak magnetic fields at nanometer length scales is a long-standing problem in physics [117], biology [118], and chemistry [119]. The NV^-^ center is a promising candidate for such a sensor owing to its remarkable magneto-optical properties [120]. The structure of the NV^-^ center is shown in Fig. 5a. Figure 5b and c illustrates the basic principle of magnetometry with the NV^-^ center by showing an example measurement in a simplified way. More in-depth studies on the spin properties can be found in [121–127]. This technique is so sensitive that the small magnetic field from a single electron spin [128] or even from a few nuclear spins could be detected [129–131]. The striking advantage of this approach over conventional magnetic resonance imaging is that one can obtain the magnetic resonance signal can by simply reading out changes in fluorescence at different microwave energies. Thus, instead of a conventional magnetic resonance imaging machine, only a confocal microscope (with a sensitive detector) and microwave electronics are needed. Additionally, the defect senses only magnetic fields which are very close by [132] (field sensitivity rapidly decreases with distance between the NV^-^ center and the sample). For review articles on the physical properties of the NV^-^ center, see [133, 134].

### Requirements for the diamond material

The sensitivity to a magnetic field is strongly dependent on the distance between the defect and the sample. To this end, researchers have two used approaches, which are both accompanied by specific problems. One strategy, which is discussed in “Reducing the size of diamond,” is to use smaller nanodiamonds. However, as already mentioned, the natural abundance NV^-^ centers in smaller diamonds is decreased. Additionally, the NV^-^ center needs to be as stable as possible and the surface chemistry should influence the sensor as little as possible. “Engineering defects” covers methods to create artificial defects close to the surface. In “Surface chemistry,” attempts to improve the properties of defects by alteration of the surface chemistry are discussed.

### Reducing the size of diamond

Centrifugation, milling, oxidation, and electrophoresis have been investigated to reduce the size of nanodiamonds. Morita et al. [135] discovered a straightforward method to reduce the size of diamond particles to 4 nm that involves simply centrifuging the nanodiamond suspension and discarding the pellet. Choosing different centrifugation speeds and times enabled them to control the size distribution of diamond particles.

Gaebel et al. [136] used oxidation in air to reduce the size of nanodiamonds. This approach has the positive side effect that nondiamond material on the diamond is removed as well. Starting with an average diamond size of 50 nm (between 0 and 100 nm), they heated the diamonds (spread on a glass slide) for 5 h and recorded the size and fluorescence at 30-min intervals. After an initial decrease in size from the nondiamond shell, they found linear etch rates of 10.6 nm/h (600 °C), 4 nm/h (550 °C), and less than 1 nm/h (500 °C) (for a comparison with other methods, see “Surface chemistry”). Say et al. [137] oxidized diamonds (0–0.1 μm) with acid to reduce the size and decrease aggregation. To this end, they refluxed diamonds for 3 days at 70 °C with a 9:1 ratio of concentrated sulfuric acid and nitric acid, followed by ultrasonication for 1 h. The procedure was repeated two times, and the nanodiamonds were refluxed for 1 h at 90 °C in a 0.1 M solution of sodium hydroxide, followed by a further 1 h at 90 °C in 0.1 M hydrochloric acid. Lastly, the nanodiamonds were thoroughly rinsed with deionized water. They further decreased the size of the nanodiamonds by oxidation in air at 600 °C for 6 h. With this method they obtained diamonds with an average size of 15 nm.

Another interesting method was described by Hens et al. [138]. They used electrophoresis to separate nanodiamonds that were of different size and functionalized differently. During electrophoresis, particles migrate with different speeds according to their different size or surface chemistry. The electrophoresis was performed for 20–45 min at 10 V. With this technique they captured different size fractions of nanodiamonds.

Boudou et al. [35] discovered a method based on milling and implantation to generate small diamonds with a high yield of fluorescing defects. They used micrometer-sized raw material that was then irradiated with electrons in the megaelectronvolt range. After it has been annealed, the diamond material is processed for days in several milling steps. Then the material is sieved to remove remaining large particles. Finally, the diamond particles are acid cleaned to provide oxygen termination. The method is quite cumbersome, but seems to have a very high yield.

One has to face a few problems when using the smallest available diamonds: small diamond size is usually paired with less stable and less abundant NV^-^ centers. Optically selecting defect centers is complicated as the graphite content is relatively large, and there are often fluorescing molecules on the diamond surface. As a result, often compromises are made, and not the smallest possible diamonds are used.

### Engineering defects

Methods to create artificial defects have been investigated since the yield of NV^-^ centers in small diamonds is decreased. There are two ways to increase the number of NV^-^ centers in diamond. One can either implant nitrogen (see “Implanting nitrogen”) or create vacancies (see “Creating vacancies”). In both cases an annealing step follows. To this end, the diamond material is exposed to a high temperature in a reducing or vacuum environment (oxygen would destroy diamond at these temperatures). During this step, vacancies are mobilized in the material. When a vacancy moves next to a nitrogen atom, it becomes trapped since it is stabler there, and the NV^-^ center is created. The same methods are also relevant for bulk diamonds because one can precisely control the proximity to the surface and the position of the defect. Which of these methods is applicable depends on the material; however, it must be critically remarked that yields in terms of number of NV^-^ centers created remain low.

#### Implanting nitrogen

Implanting nitrogen is the best way to create NV^-^ centers in terms of performance. Furthermore, the method can be used in very pure diamond, which is favorable since less background from impurities can be expected. The performance can be improved even further by use of isotopically purified ^12^C diamond (naturally abundant ^13^C also interferes with the NV sensor) [4, 139]. The position and depth of the defect can be precisely controlled. Notably, Pezzagna et al. [140] generated NV^-^ centers with a special resolution below 20 nm by implantation through a mica mask.

Ofori-Okai et al. [141] obtained NV^-^ centers very close to the diamond surface by using low energies for implantation. Table 1 summarizes different conditions that were reported in the literature for implantation of nitrogen. For comparison, the conditions and the most important findings (including the methods they were based on) are listed. For very pure diamonds, where the nitrogen content limiting, implantation with nitrogen is necessary. In high-pressure, high-temperature diamonds or other diamond sources where nitrogen is relatively abundant, vacancies can be created alternatively.

**Table 1:** Summarized conditions for nitrogen implantation and the outcome of experiments (and how it was evaluated)

Diamond sample	Irradiation	Energy	Annealing	Conclusion	Reference
Bulk CVD diamond	N ions (10^10^–10^11^/cm^2^)	10–50 keV	1 h, 900 °C, Ar/H_2_ (then O_2_ annealing 465 °C)	Fluorescence increased (PL)	[143]
Bulk, single crystal	^15^N^+^, ^15^N^2+^	0.4–5 keV	2 h, 800 °C, vacuum (then acid reflux)	More NV^-^ centers (PL, ODMR)	[141]
Ultrapure bulk diamond	N^+^	2 MeV	1 h, 800 °C, vacuum	NV^-^ centers are created	[140]
Ultrapure bulk diamond	^15^N_2_	2–5 keV	2 h, 800 °C, vacuum	NV^-^ centers are created (ODMR)	[182]

*CVD* chemical vapor deposition, *NV* nitogen vacancy, *ODMR* optically detected magnetic resonance, *PL* photoluminescence

#### Creating vacancies

**Fig. 6:**
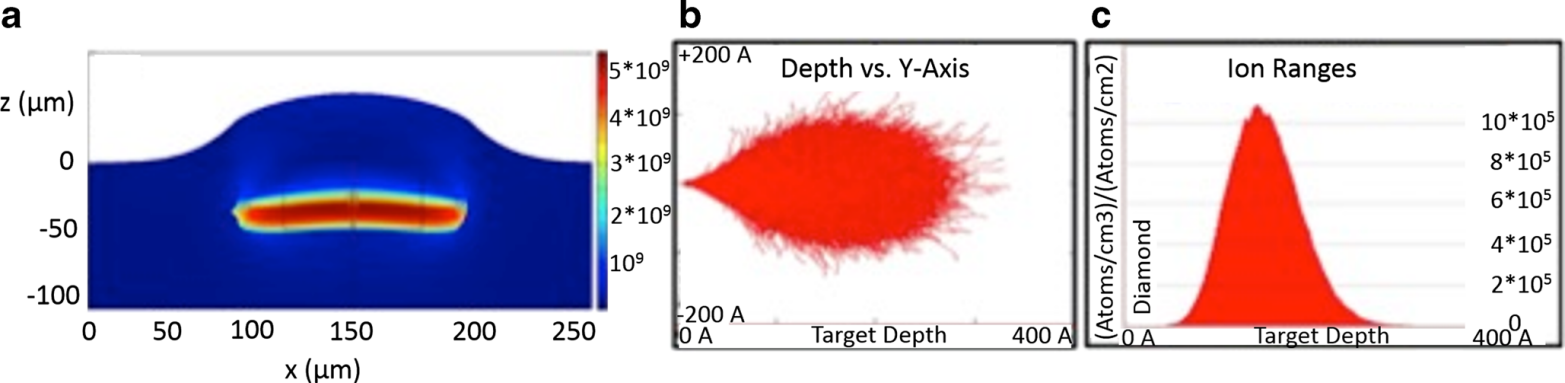
Damage from implantation. **a** The deformation and the damaged area after a 3-MeV hydrogen implantation. **b** The trajectory profile and **c** the range of 30-keV Ga ions in diamond simulated by SRIM. The projected range is about 15 nm and the lateral straggling is 4 nm. (**a** Reprinted with permission from [180]; **b**, **c** reprinted with permission from [181])

The most prominent way to create vacancies is to irradiate diamond with ions. This process has been theoretically investigated by Deák et al. [142]. Experimentally, several different ions and conditions have been used, and these are summarized in Table 2. During irradiation, vacancies are created throughout the material until the ion finally stops. At which depth it stops depends on the energy the ion had. An example of irradiation with gallium is shown in Fig. 6b and c.

**Table 2:** Summary of different methods to create vacancies by atom bombardment

Diamond sample	Irradiation	Energy	Annealing	Instrument	Conclusion	Reference
Detonation NDs (and ND in PDMS)	H^+^ (10^12^–3.2 × 10^16^/cm^2^, best at 3.2 × 10^16^/cm^2^)	2 MeV (50 nA)	1 h, 600 °C, vacuum	Tandetron accelerator	Fluorescence increases	[182]
Bulk	Ga^+^	30 keV (3 pA, 3 ms)	1 h, 750 °C, vacuum	Orsay FIB 3000	Fluorescence increases (PL)	[183]
Bulk CVD diamond	Ga ions (10^12^–10^15^/cm^2^)	30 keV	5–120 min, 300–900 °C (30 min, 900 °C required to recover NVs)		Fluorescence of NV^-^ disappears but is recovered after annealing or being subjected to H_2_ plasma for 15 min (CL)	[144]
Bulk type IIa diamond	B ions (2 × 10^16^/cm^2^)	60 keV (1 uA/cm^2^)	1 h, 1100 °C, low vacuum (Ar, H_2_)		Vacancies created (SIMS)	[147]
Bulk type IIa diamond	As ions (2 × 10^16^/cm^2^)	320 keV	1 h 1100 °C, low vacuum (Ar, H_2_)		Vacancies created (SIMS)	[147]
Bulk HPHT single crystal	Si ions (10^14^–10^15^/cm^2^)	1 MeV	10 or 40 min 950 °C N_2_	Tandem accelerator ion implanter	Vacancies are created during implanting (TEM)	[145]
Bulk type Ib diamond	H^+^ (10^12^–10^17^/cm^2^, optimum 5 × 10^16^/cm^2^)	2.4 MeV	1–20 h, 600–1000 °C (optimum 20 h, 800 °C)	Van de Graff accelerator	Number of NV centers is increased to 2.3 × 10^18^/cm^3^ (PL, PAS)	[184]
HPHT diamond	He ions (10^6^-10^17^/cm^2^)	1.8 MeV (1 nA)	1000 °C	Ion microbeam line INFN	Vacancies and graphitization (simulation, XRD)	[185]
CVD diamond IIa	B ions (10^13^/cm^2^)	180 keV	1000 °C	Olivetti I-Jet facilities	Vacancies and graphitization (simulation, XRD)	[185]
100-nm ND	H^+^ (5 × 10^15^/cm^2^)	3 MeV	2 h, 800 °C, vacuum	NEC tandem accelerator	Fluorescence increased no bleaching (PL)	[55]

*CL* chemiluminescence, *HPHT* high-pressure, high-temperature, *ND* nanodiamond, *PAS* positron annihilation spectroscopy, *PDMS* polydimethylsiloxane, *SIMS* secondary ion mass spectrometry, *TEM* transmission electron microscopy, *XRD* X-ray diffraction

There are three main difficulties that one needs to take into account in ion irradiation. First, if the implanting ions remain in the diamond, they might alter the properties of the NV^-^ center [142 ,143]. Second, irradiation of the substrate (e.g., glass) might lead to an increased background fluorescence. Third, too high ion doses lead to unwanted damage of the diamond crystal structure [145]. Up to a certain amount of damage in the crystal structure can be regenerated during annealing, but above a certain threshold, the damage is irreversible. Several authors determined that threshold to be around 10^14^ to 10^15^ ions per square centimeter [146]. This effect was found to be almost independent of the ion type and implantation energy [147]. Implantation damage as well as contamination with ions can be circumvented when vacancies are created by irradiation with electrons or protons. However, since these are so small, very high voltages and high doses (and thus expensive equipment) are needed to create defects. Table 3 summarizes different conditions that were used to create vacancies with elementary particles. Despite all these efforts, generating NV^-^ centers with high yield without damaging the crystal structure remains a major limitation.

**Table 3:** Methods for creating vacancies with elementary particle irradiation

Diamond sample	Irradiation	Energy	Annealing	Conclusion	References
Bulk	Electrons	400 keV	1 h, 750 °C, vacuum	Fluorescence increases (PL)	[183]
Bulk type Ia, IIa, and Ib diamond	Electrons	2 MeV	0–22 h, 600–800 °C, vacuum	Fluorescence increases (PL)	[55, 186]
ND (powder, 50 nm)	Electrons	13.9 MeV (2–60 min)	2 h, 800 °C (then piranha solution cleaning)	Each diamond is fluorescent (PL)	[187]
Micrometer-sized diamond powder	Electrons	10 MeV (8 mA)	800 °C, vacuum (then milling)	More NDs contain NVs (ODMR, PL)	[35]
Bulk type Ib diamond	Neutrons (0.7 × 10^16^–2.8 × 10^18^/cm^2^)	0.1–7 MeV	1 h, 900 °C	NVs are created until a certain dose, then NV^0^ (PL)	[188]

### Surface chemistry for magnetometry applications

**Fig. 7:**
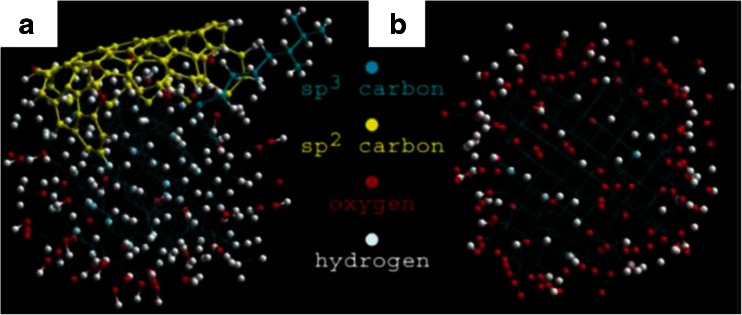
Structural model for diamond with graphitic (*sp* ^2^) and other nondiamond surface defects (**a**) and oxygen-terminated nanodiamond (**b**). (Reproduced with permission from [157])

Whereas for biolabeling applications the diamonds should be as bright as possible, for magnetometry it is also crucial that the stability of the defect is guaranteed. In bioapplications, for example, hydrogen-terminated diamonds are often preferred since the surface chemistry is more uniform. In contrast, for magnetometry applications, where the defect center should be close to the surface, δ^+^ hydrogen termination is less preferred, whereas oxygen termination results in increased luminescence (compared with hydrogen termination) [148, 149], and even conversion of NV^−^ centers to uncharged NV^0^ centers was observed [150]. Another problem is paramagnetic defects on the surface [151] or in the material since they alter the lifetime of the NV^-^ center. Oxygen from air [152] and internal defects from diamond synthesis [153] (most prominent in detonation nanodiamonds) or surface treatment [154] are possible causes of these defects. These effects have been systematically investigated by Rosskopf et al. [151]. The NV^-^ center can lose its charge, and thus cannot be used anymore [155]. Consequently, electronegative termination (as oxygen or fluorine) is more popular in magnetometry. However, for fluorine termination, bleaching effects were observed [33, 130]. Another major problem is graphite on the surface (for a structural model, see Fig. 7). Graphite molecules on the surface are unfavorable since the surface properties are difficult to control. Graphite (or other unsaturated carbon compounds) can react with NV^-^ centers and form NV^0^ instead of NV^-^, which has the desired optical and spin properties [143]. Furthermore, graphite quenches the fluorescence and thus reduces the counts, especially for NV^-^ centers close to the surface [156]. Organic molecules on the surface often also cause increased background fluorescence. Methods for reducing the graphite content of nanodiamonds are all based on oxidation of the outer shell of nanodiamond. Possible oxidation methods to achieve this goal are acid cleaning, high temperature [136, 143, 157], or UV/ozone cleaning. Whereas acid cleaning and air oxidation below 400 °C are used for selective oxidation of the nondiamond shell, UV/ozone and air oxidation at higher temperatures sacrifice the outer diamond layer.

### Attaching diamond to nanostructures

To use diamonds in a magnetometry experiment, one has to move a sample relative to the NV^-^ center. To this end, one can scan a sample relative to an NV^-^ center and record the optically detected magnetic resonance [158]. This can be done by attaching the sample to an atomic force microscope (AFM) tip. The fact that the sample has to be attached to an AFM tip limits the approach to very small samples (nanometers to micrometers). An elegant alternative is to attach the diamond to an AFM tip. This was independently proposed by Degen [159] and Taylor et al. [160] and experimentally realized by Rondin et al. [161]. To achieve that goal one can either use tips that are fabricated from bulk diamonds [162] or attach nanodiamonds to a tip. Diamond tips have the advantage that bulk diamond material of high purity or with a preferred orientation [163] can be used and the structure can be fabricated to increase light collection efficiency (waveguide effect). However, the microfabrication of these structures is challenging and time-consuming. Additionally, engineering defects at the very end of the diamond structure and thus close to the sample is also not trivial. Nanodiamonds tend to have worse magneto-optical properties but are easier to use and owing to their small size the defect is always close to the surface. There are a few methods available to attach nanodiamonds to standard AFM tips. In the very first experiments, the diamond particle was simply glued to the AFM tip [164, 165]. An improvement of that technique was to use polylysine, which is a positively charged peptide, for attachment. In that method the diamond particle is attached simply by electrostatic interactions between positively charged groups on polylysine and negative charges on the surface of oxygen-terminated diamond [161].

### Limitation for magnetometry applications

The main drawback for this application is that the diamond has to be close to the sample. As a result, only the very surface of a sample can be investigated or the probe has to be inserted in the sample, which might alter the natural behavior. Additionally, if the nanodiamonds have to be inserted into the sample, it has to be at least to some extent transparent. And the diamond has to be close enough to the objective to allow focusing. Especially when working with single emitters, one has to take care to avoid background fluorescence. Working with multiple emitters, on the other hand, broadens spectral lines. Furthermore, the need to use a laser and microwaves leads to warming of the sample, which might be problematic for some applications and thus require active cooling or temperature stabilization. Lastly, surface terminations with electropositive groups too close to the defect might cause the defect to lose its charge and thus its superior optical properties. This limits the possibilities for surface termination of the diamond particle.

## Conclusion

Diamond has various extreme properties making it an outstandingly promising candidate for many different applications. Intense research in surface and defect chemistry has improved diamond quality significantly. However, there is still much room for improvement in this very new field. In particular, optimizing surface chemistry, improving defect yield, and simultaneously reducing the size and preventing aggregation are still issues. Systematic research on the uptake of nanodiamonds in cells (especially as a function of the surface chemistry) for in vivo use is scarce. Likewise long-term toxicity and the fate of nanodiamonds within cells are poorly understood; thus, in vivo biolabeling still has major obstacles that need to be overcome. Furthermore, several new material properties have been identified which can be detected with defects in diamond. Among these are temperature (also in vivo) [166–169], strain [170], pressure [171], orientation [172], and electric fields [173]. Thus, imaging these quantities might lead to entirely new imaging modes and applications.

Access to temperature measurements with a spatial resolution on the order of a few nanometers could make possible applications such as temperature-induced control of gene expression, cell-selective treatment of disease, and the study of heat dissipation in integrated circuits [168]. For measurements of the orientation of fluorescent nanodiamonds, we may expect insight into electromagnetic sensing of the cellular environment, such as in neuronal networks, ion channel activity, and embryological development [172]. Detecting electric fields on the nanoscale, on the other hand, may proved to be valuable tool in the development of quantum devices or bioimaging. [173]. Therefore, we may expect to see exciting results and applications emerging in this field.
